# Where in the genome are we? A cautionary tale of database use in genomics research

**DOI:** 10.3389/fgene.2013.00038

**Published:** 2013-03-21

**Authors:** Laura K. Vaughan, Vinodh Srinivasasainagendra

**Affiliations:** Section on Statistical Genetics, Department of Biostatistics, University of Alabama at BirminghamBirmingham, AL, USA

**Keywords:** reproducible research, database, bioinformatics, gene mapping

## Abstract

With the advent of high throughput data genomic technologies the volume of available data is now staggering. In addition databases that provide resources to annotate, translate, and connect biological data have grown exponentially in content and use. The availability of such data emphasizes the importance of bioinformatics and computational biology in genomics research and has led to the development of thousands of tools to integrate and utilize these resources. When utilizing such resources, the principles of reproducible research are often overlooked. In this manuscript we provide selected case studies illustrating issues that may arise while working with genes and genetic polymorphisms. These case studies illustrate potential sources of error which can be introduced if the practices of reproducible research are not employed and non-concurrent databases are used. We also show examples of a lack of transparency when these databases are concerned when using popular bioinformatics tools. These examples highlight that resources are constantly evolving, and in order to provide reproducible results, research should be aware of and connected to the correct release of the data, particularly when implementing computational tools.

## Introduction

When conducting genetics research, whether from the perspective of a candidate gene study or genome wide association study (GWAS), researchers must be able to accurately identify and translate where molecular markers are located on the genome in reference to the coordinates of known genes. While this may seem straightforward, it can be quite complicated and is often overlooked (Hong et al., 2009; Wang et al., 2010). The mapping of markers to genes and subsequent data mining of information about these genes is further complicated by the ever increasing amounts of data and resulting evolution in databases, which in turn can lead to changes in genomic coordinates, annotations, and other information. Additionally, few studies (and methodologies) report the version of databases that are used in the bioinformatic workflow process. For example, the popular bioinformatics resource DAVID lists the download date of the various databases used for the Knowledgebase (the most recent of which is in 2009), but does not provide the version of those databases (Huang et al., 2009a,b). This lack of reporting can make subsequent analysis and reproduction of others research difficult, if not impossible. In this manuscript we describe the key steps involved in the use of database resources for the mapping of markers to genes (and vice versa) in a typical candidate gene based study and highlight several ambiguities that can have potentially serious consequences in subsequent research.

## Workflow case studies

The steps in identifying SNPs from a list of candidate genes can be described as (1) determining the candidate gene pool, (2) annotating, or retrieving information about those genes, (3) determining the location (and boundaries) of those genes, and (4) identifying molecular markers (e.g., single nucleotide polymorphisms, SNPs) within those boundaries. Similar steps are involved in identifying genes that are related to SNPs found to be significant from a GWAS.

In a collaborative research setting investigators will often provide a list of gene names that they are interested in to their bioinformatic collaborators who then retrieve information related to these genes for further analysis. The naming of these genes is the first point of ambiguity. Often, these names are common names or synonyms instead of the official names or gene symbols [see HUGO Gene Names Committee HGNC (Seal et al., 2011)]. Due to the structure of most bioinformatic data sources, it can be difficult to correctly identify the gene that an investigator is interested in when the official name is not provided. An example of this is described in Table 1. In this example the original list of *TOSO*, *PIGR*, *FCAMR*, *ADRA1A*, *ADRA1B*, and *ADRA1D* was provided by a collaborator. When searching the UCSC Genome Browser or Entrez Gene databases (accessed July 2011), we see that *TOSO* is not an HGNC official gene symbol, but is instead a synonym for the gene Fas apoptotic inhibitory molecule 3 (official symbol *FAIM3*, geneID 9214), and searching earlier versions of UCSC Genome Browser or into the Gene Accession Conversion tool in DAVID, *TOSO* would not produce any results.

**Table 1:** **Inconsistency in gene “names” and locations**.

**GeneID**	**Symbol**	**Synonyms**	**Chr**	**Description**	**Genome build/base pair location**		
					**Hg17**	**Hg18**	**Hg19**
9214	*FAIM3*	**TOSO**	1	Fas apoptotic inhibitory molecule 3	203466126-203483738	205144354-205161966	207076633-207095378
5284	***PIGR***	FLJ22667|MGC125361|MGC125362	1	Polymeric immunoglobulin receptor	203490267-203508202	205168495-205186430	207101869-207119811
83953	***FCAMR***	FCA/MR|FKSG87	1	Fc receptor, IgA, IgM, high affinity	Information Not available	205198027-205210593	207131404-207143970
148	***ADRA1A***	ADRA1C|ADRA1L1|ALPHA1AAR	8	Adrenergic, alpha-1A-, receptor	26683139-26778839	26683139-26778839	26627222-26722922
147	***ADRA1B***	ADRA1|ALPHA1BAR	5	Adrenergic, alpha-1B-, receptor	159276318-159332129	159276318-159332595	159343740-159400017
146	*ADRA1D*	ADRA1|**ADRA1A**|ADRA1R|ALPHA1|DAR|dJ779E11.2	20	Adrenergic, alpha-1D-, receptor	4149816-4177659	4149278-4177659	4201278-4229659

*Bold genes indicate terms from original list from collaborator. Annotation information retrieved for the candidate gene list through Entrez gene's GeneInfo. GeneInfo data can be downloaded from Entrez gene's FTP location on September 10th 2010 (ftp://ftp.ncbi.nih.gov/gene/DATA/)*.

The second source of ambiguity, gene annotation, is also illustrated in Table 1. In general, capturing gene level annotations (HGNC id, geneID, synonyms, chromosome, description, etc.), not only provides more information, but also allows investigators to perform quality checks. In the example discussed here, *ADRA1A* is listed as both an HGNC official gene symbol (adrenergic alpha-1B-receptor, gene ID 147) and as a synonym for *ADRA1D* (adrenergic alpha-1D-receptor, gene ID 146). Without scrutiny, it is difficult to tell which gene(s) the investigator is indeed interested. A survey of the most recent build of Entrez Gene (Hg 19) reveals that there are 43,037 unique gene symbols, 53,215 unique gene synonyms and 1122 instances where a term is both an official gene symbol and a synonym for at least one other gene, and 2632 terms that occur as synonyms for multiple genes, Although it may seem trivial in this example where there are only a few genes, in situations where there are dozens to hundreds of genes this manual verification of genes represents a significant investment of time and potential sources of error. It is also important to note that inconsistencies between databases can also introduce significant errors when translating gene IDs from one source to another. Even when using one of the several ID converters available (e.g., DAVID ID Converter or GeneCruiser), errors can be introduced when synonyms, HGNC symbols and other identifiers are inconsistently mapped or when the timelines for the database releases are not correctly matched or are out of date (discussed further below).

In the candidate gene framework, the gene coordinates can be identified from databases such as Entrez Gene or UCSC Genome Browser relatively easily. However the third ambiguity, determining the gene location, is illustrated in part by inconstancies in the use of gene symbol vs. gene synonym (Table 1). For example, the genes *ADRA1A* and *ADRA1D* (discussed above) are located on separate chromosomes. Choosing the wrong gene will result in choosing a completely inappropriate location which will have obviously significant potential implications on downstream analysis. This ambiguity is perhaps more of a concern when taking the approach common for GWAS of identifying genes related to interesting SNPs. For a GWAS, usually both the SNP coordinates and genes that contain those SNPs are provided by the manufacturer of the genotyping platform. However, how these coordinates and genes are identified is often unclear, and these annotation files themselves are often additional source of errors. This is strikingly illustrated in Table 2. In this instance a SNP (rs2844871) was identified as interesting based on an association study genotyped on the Affymetrix Genome Wide Human SNP Array 6.0. When following the bioinformatic workflow to identify the gene of interest, it was discovered that the SNP is mapped to different genes based on not only different databases, but also on different versions of those databases. A query of build 135 of dbSNP identified 1,226,430 SNPs that have multiple coordinates, 805,555 of which have more than one distinct chromosome assigned to the same rs ID (or 1.5% of the 54,212,080 SNPs). Additionally, 1,164,480 single base pair coordinates were found to be associated with multiple rsID's (with the maximum of 97 rsID's associated with the coordinates for one single nucleotide polymorphism). Searching for annotation information for rs2844871 in the UCSC Genome Browser, dbSNP, HapMap and Affymetrix databases not only provided different genomic locations based on which build that was accessed, but different (and multiple) chromosomes. Although in this case the multiple locations are likely due to a duplication event [a BLAST (http://blast.ncbi.nlm.nih.gov/) search of the 100 base pair sequence surrounding the SNP shows that regions of >90% identity occur on chromosomes 22, 14, 2, 4, and 21], it serves to dramatically illustrate errors that can be introduced with the use of different databases and lack of stringent quality controls.

**Table 2:** **Discrepancies in the location of SNP rs2844871**.

**Database**	**Database version**	**Human genome build**	**Human reference chromosome/NCBI build**	**dbSNP build**	**Location (bp)**
Affymetrix 6.0	NetAffx 30	Hg18	NCBI36	Not specified	chr22:14459243
	NetAffx 31	Hg19	GRCH 37	131	SNP is listed, but no position information given
	NetAffx 32	Hg19	GRCH 37	132	SNP is listed, but no position information given
UCSC genome browser	July 2011	Hg18	NCBI36	128	chr22: 14459242
		Hg18	NCBI36	130	chr14: 19763716
					chr22: 16079242
		Hg 19	GRCH 37	132	chr14:19763467
					chr22:16078993
					chrUn_gl000244:34403
		Hg 19	GRCH 37	135	chr14:19763467
					chr22:16078993
					chrUn_gl000244:34403
dbSNP	July 2011	Hg 19	NCBI 37.1	132	chr2: 125655701
	October 2012	Hg19	NCBI 37.3	137	chr14: 19763717
					chr22: 16079243
					NA
					chr2:124523528
HapMap	Release 27	Hg18	Not specified	Not specified	chr22: 14459243^*^
HapMap	Release 28	Hg18	NCBI36	126	chr22: 14459243

* *Genotyped on Affy 6.0 for Phase II samples, no dates or other information was given in HapMart*.

*Search results for SNP rs284487 from various sources. Search conducted on Jul 11th, 2011 and updated October 29th, 2012*.

Additionally, when mapping a marker to a gene, investigators are often not just interested in a SNP that lies directly with the gene boundary, but also genes that lie within a certain distance or are in linkage disequilibrium with a SNP of interest. The accurate identification of SNPs and related genes is dependent on both an accurate identification of gene boundaries and the synchronization of multiple databases, which often leads to the final source of ambiguity and is discussed further below.

The final source of ambiguity, variation between databases and across time, is intrinsic to every step of the workflow outlined above. Bioinformatic analysis is dependent on key database resources such as dbSNP, Entrez Gene, UCSC Genome Browser and Ensembl (Sherry, 2001; Fujita et al., 2011; Maglott et al., 2011; Flicek et al., 2012). These databases are in a state of dynamic flux, and are constantly being updated, sometimes resulting in significant changes (Data Changes that Occur Between Builds, 2005; Fujita et al., 2011). More often than not, investigators fail to provide the date and database version of each of the data sources that was used in the process of their analysis. Comparing the number of official gene symbols and synonyms for Hg18 and Hg19 highlights the differences in database builds. As discussed above, there are 43,037 and 53,215 unique gene symbols and synonyms respectively in Hg19, compared to 38,586 and 53,475 in Hg18, with 23,325 gene symbols overlapping between the two versions of the human genome builds. Tables 1 and 2 illustrate how the version of the database used can have an effect on the data that is retrieved. For example, (1) *FACMR* (gene ID 83953) was not included in the human genome build 17 (Hg17) and has gene boundary location that is shifted by almost two million bases from Hg18 to Hg19 and (2) when the coordinates from Hg17 are used to search UCSC Genome Browser using Hg19 the gene *OPTC* (gene ID 26254) is retrieved instead of *FAIM3*. Although these shifts in boundaries are a result of updates to the genome builds, one can see how using gene boundary coordinates from Hg19 for data that was originally built on Hg17, without first correcting for the change, can introduce errors. Tools such as the UCSC Genome Browser LiftOver Utility are available to convert genome coordinates between assemblies; however to correctly apply the tool, researchers must first be aware of the issue.

Furthermore, the timeline of these changes is not coordinated across databases. A search conducted in January 2010 in dbSNP would have been based on Hg18 instead of Hg19 which the NCBI released in early 2009 (build #37) and the August 2010 HapMap data release (release #28) for both Phase II and III data is based on NCBI build 36 and dbSNP release 126 (from 2006). As discussed above, the location of a gene may change with different builds of the human genome, sometimes significantly, and investigators should take the necessary steps to ensure that they are using coordinated builds of the different resources. When conducting research that is based on a genotyping chip, investigators should also carefully consider the version of databases used for bioinformatic analysis. If the corresponding changes in the coordinates of the markers on the genotyping chip are not also accounted for, SNPs could be mapped to incorrect genes, which can result in very costly mistakes (Karow, 2010).

## Discussion

In recent years there has been a paradigm shift in the field of genetics. In the not too distant past, researchers were limited by their ability to acquire data. Now, with the availability of genome scale DNA and RNA platforms and recent introduction of affordable whole genome sequencing technologies, scientists are limited by their ability to effectively organize and analyze vast amounts of data. Part of this process is the accurate and consistent annotation of genomic information as part of the bioinformatics workflow. As describe above, changes in database versions and genome builds throughout the life of a study can have potentially significant impact.

In the case study discussed here we highlight several ambiguities that can be introduced in a candidate gene or SNP based study. When going across database versions using gene name, coordinates or rsID's up to the individual researcher. In the candidate gene based approach where the aim is to identify variants within a gene, one will typically use the coordinates of both genes and SNPs to identify SNPs for further study. As described above, one first verify they have the correct gene, and then must either stay within the same human genome version for each database used, or must correctly convert coordinates in order to avoid introducing errors. For the complementary approach based on identifying genes related to interesting SNPs, often the only data provided is the rsID for that SNP and no coordinates or genome build information is provided. Without this extra information errors can again be introduced when, as shown above, multiple positions, and therefore multiple genes are associated with a variant.

One way to prevent these errors is for investigators to involve bioinformaticians in all stages of a study, and for everyone involved to follow the principles of reproducible research. Reproducibility in research has been defined by the uniform Guidelines of the International Committee of Medical Journal Editors as the responsibility of authors to “identify the methods, apparatus and procedures in sufficient detail to allow other workers to reproduce the results.” Young scientists are taught to include in the methods and materials section of manuscripts the details which would be needed for successful repetition and extension of their work (Hothorn and Leisch, 2011). Unfortunately, the same attention that is given to laboratory based experimental details and protocols have not been applied to the bioinformatics or computational components of many large genetic studies. This is beginning to change, especially in the domains of bioinformatics and biocomputing, where there has been growing interest in following the philosophy and best principles of reproducibility and repeatability in scientific research (Hothorn et al., 2009; Mesirov, 2010). As we move toward fully embracing the concepts of reproducible research, there is an increasing need for reproducible research modules in many of the software and tools where underlying computer code and data tend to change over time.

The continued growth in data volume has introduced a new set of issues that must be considered and addressed in genomics studies. The examples discussed above illustrate the importance of involving bioinformaticians in the entire process of a study. Researchers can avoid these pitfalls by implementing procedures that follow the principles of reproducible research. Similar to the use of a notebook in a wet lab, a wiki based notebook (our own group uses a Confluence powered wiki), employing a Reproducible Research Systems (RSS) approach or using tools such as myExperiment, GenePattern GRRD, Galaxy or Sweave, can be used to detail the workflow involved in the computational analysis of complex genomic data (Friedrich Leisch, 2002; Reich et al., 2006; Goble et al., 2010; Goecks et al., 2010; Hothorn and Leisch, 2011). Accurate depiction of the research process will become even more important as journals follow the trend set by *Biometrical Journal*, *Journal of Epidemiology*, and *Biostatistics* which now suggest that authors go beyond the common practice of making data freely accessible, but also meet some standard of reproducibility (Peng et al., 2006; Peng, 2009; Mesirov, 2010).

## Conclusion

The importance of following the principles of reproducible research has been recently highlighted with several high profile examples (Hothorn et al., 2009; Baggerly and Coombes, 2011). Seemingly small mistakes can have significant downstream consequences in any data analysis that utilizes large amounts of data and multiple steps of analysis. As exemplified here, the simple mistake of not reporting, or using an incorrect version of a database can affect the interpretability and reproducibility of a study. To prevent these issues from having a greater impact, it is important for the research community as a whole to embrace the concepts of reproducible research and make a conscious effort toward moving toward that goal.

### Conflict of interest statement

The authors declare that the research was conducted in the absence of any commercial or financial relationships that could be construed as a potential conflict of interest.

## Figures and Tables

**Table:** 

**Resource**	**URL**
Entrez Gene	www.ncbi.nlm.nih.gov/gene
UCSC Genome Browser	http://genome.ucsc.edu
Affymetrix NetAffx Analysis Center	www.affymetrix.com/analysis/index.affx
dbSNP	www.ncbi.nlm.nih.gov/projects/SNP/
UCSC LiftOver	http://genome.ucsc.edu/cgi-bin/hgLiftOver
My Experiment	www.myexperiment.org/
GenePattern GRRD	www.broadinstitute.org/cancer/software/genepattern/grrd/
Sweave	www.stat.uni-muenchen.de/~leisch/Sweave/
HUGO	www.genenames.org/aboutHGNC.html
DAVID	http://david.abcc.ncifcrf.gov/
GeneCruiser	http://genecruiser.broadinstitute.org/genecruiser3/
